# The impact of rural tourism entrepreneurs' hometown identity on entrepreneurial persistence

**DOI:** 10.3389/fpsyg.2025.1513420

**Published:** 2025-02-05

**Authors:** Fuhua Xiang

**Affiliations:** School of Management, Zhejiang University, Hangzhou, China

**Keywords:** rural tourism entrepreneurs, hometown identity, entrepreneurial persistence, theory of planned behavior, Guizhou Province

## Abstract

**Introduction:**

This study explores how hometown identity of rural tourism entrepreneurs affected entrepreneurial persistence.

**Methods:**

Data from 494 rural tourism entrepreneurs were collected using convenience sampling and analyzed using Amos and SPSS statistical software package via questionnaires distributed to three villages in Guizhou province, China.

**Result:**

The findings reveal that (1) rural tourism entrepreneurs' hometown identity positively affected their attitudes toward entrepreneurial persistence and indirectly influenced entrepreneurial persistence; (2) rural tourism entrepreneurs' hometown identity positively influenced perceived behavioral control and indirectly affected entrepreneurial persistence; (3) rural tourism entrepreneurs' hometown identity positively moderated the role of subjective norms on entrepreneurial persistence.

**Discussion:**

The findings from the present study indicated that rural tourism entrepreneurs' hometown identity can shape entrepreneurial persistence through numerous means. Authorities should emphasize the cultivation of a strong hometown identity among entrepreneurs when formulating and implementing destination-related policies to ensure the sustainable development of rural tourism.

## 1 Introduction

Tourism entrepreneurship has been increasingly regarded as a critical tool for rural revitalization in developing countries such as China (Sun et al., 2022). Many rural families have launched businesses in hopes of earning a better livelihood (Wu et al., 2020). Yet entrepreneurship is an inherently high-risk endeavor featuring diverse challenges (Kim and Seo, 2019). This precariousness has been especially pertinent over the past 3 years: tourism entrepreneurs faced unprecedented obstacles (Coppens and Knockaert, 2021; Eli, 2021) as the COVID-19 pandemic swept the global tourism industry (Islam et al., 2022). For example, domestic tourist visitation plummeted by 52.1% in 2020 (Finance Department of the Ministry of Culture Tourism, 2021). Such circumstances have greatly undermined tourism entrepreneurs' work-related confidence, with a large number of entrepreneurs abandoning their businesses (Islam et al., 2022). Other entrepreneurs have opted to stay in business despite the crisis. Their persistence represents a valuable force behind rural tourism recovery in the post-pandemic era. As such, nurturing rural tourism business owners' entrepreneurial persistence is critical for both personal success and rural tourism destinations' sustainable development. Therefore, the purpose of this paper is to examine the factors that shape the persistence of rural tourism entrepreneurs.

Entrepreneurship is an enduring topic in the tourism literature, with businesses' start-up and survival having gained particular attention. Studies on business openings generally pertain to entrepreneurial intentions. Scholars have identified numerous influencing factors: entrepreneurial traits (An and Craig, 2023; Saeed et al., 2023), entrepreneurship education (Kusumawardani et al., 2020; Maryetti et al., 2019), external support (Boi and Ngoc, 2019; Kang, 2022; Tamer et al., 2022; Zhang and Li, 2019), and the entrepreneurship environment (Luo, 2021; Patrick et al., 2024). Similarly varied factors have been found to inform businesses' survival. Examples include the regional environment and competitive advantage (Cheng et al., 2022; Kemal and Hilal, 2019), business management strategies (Siwarit et al., 2021), and entrepreneurs' characteristics (Brouder and Erikssion, 2013; Sanchez-Medina et al., 2019). Meanwhile, little work has examined tourism business owners' entrepreneurial persistence. The psychological process guiding these individuals' ongoing pursuit of entrepreneurship and survival thus remains unclear.

Entrepreneurial persistence represents a pertinent topic in mainstream entrepreneurship research. Authors have studied this notion on the bases of entrepreneurs' attributes (Baum and Locke, 2004; Caliendo et al., 2014; Freeland and Keister, 2016; Patel and Thatcher, 2014), environmental factors (Haines and Townsend, 2014; Hayward et al., 2006; Mujtaba et al., 2021; Steel and Konig, 2006; Welter and Smallbone, 2011), and psychological dimensions (Benjamin and Hess, 2011; Chen et al., 2021; Elhakim, 2022; Van Scotter and Garg, 2019; Silla, 2020). Despite these efforts, several knowledge gaps call for further attention. First, because entrepreneurial persistence is partly an emotional process, more needs to be uncovered about emotional factors and their effects on persistence (Lv et al., 2017; Shepherd, 2003). Second, the extant literature largely reflects a Western cultural perspective. Compared with this cultural focus on rationality and speculation, Confucianism has influenced entrepreneurs in many Eastern countries—especially China—since childhood; their entrepreneurial persistence may thus differ from that of their Western counterparts (Li et al., 2021; Liu, 2020). The novelty of this paper lies in its exploration of the mechanisms through which affective factors influence entrepreneurial persistence, set against the backdrop of Chinese cultural context, thereby bridging a gap in current research.

In Confucian culture, entrepreneurs' emotional connections with their hometowns are readily apparent. These business owners tend to be bonded to the places where their ancestors and childhood memories reside (Chen et al., 2020). Such complex person—place links are also termed “*Xiang Zi Zhi Qing*” (乡梓之情). The idea of “hometown identity” profoundly shapes Chinese entrepreneurs' behavior (Huang et al., 2023; Sun et al., 2022; Zhuang and Chen, 2018). This phenomenon is prevalent in rural tourism; because these entrepreneurs are mostly aborigines, their products reflect their traditional lifestyles. Even so, researchers have rarely explored whether and how hometown identity may shape entrepreneurial persistence.

To fill the aforementioned knowledge gaps, the construct of hometown identity was adopted in this study to explain the entrepreneurial persistence of rural tourism business owners in the Chinese countryside. First, a conceptual model was developed to illustrate the relationships among hometown identity, attitudes, subjective norms, perceived behavioral control, and entrepreneurial persistence. Second, the paper details the methodology, encompassing instrument design and measurement, study sites, data collection, and data analysis. The model was subsequently tested using data from 494 responses collected from three rural tourism destinations in Guizhou Province, China: Xijiang Miao Village, Zhaoxing Dong Village, and Nahui Buyi Village. All three villages are home to ethnic minority communities that have successfully preserved their traditional culture and way of life. Renowned as prominent rural tourism destinations in Guizhou Province, China, they attract numerous entrepreneurs, the majority of whom are locals. These entrepreneurs place great significance on local culture and possess a deep connection to their region. Finally, the findings and their implications are presented.

## 2 Literature review

### 2.1 Entrepreneurial persistence

To realize the economic benefits of their entrepreneurial activities, individuals must not only become entrepreneurs but also persist in their business ventures (Chen et al., 2021). Entrepreneurial persistence captures business owners' tendency to withstand adversity, even in the face of risk (Zhang, 2018). Entrepreneurial persistence implies steadfast adherence to entrepreneurial objectives and actions (Coppens and Knockaert, 2021). Because enterprise creation is accompanied by uncertainty, persistence is arguably the most valuable entrepreneurial quality (Elhakim, 2022) and is the cornerstone of business owners' success (Huang et al., 2023; Randolph et al., 2022; Sabiu et al., 2018). People who are persistent may nevertheless struggle to navigate constraints in today's competitive business environment (Afsaneh and Ying, 2023; Van Scotter and Garg, 2019).

Entrepreneurship scholars generally agree that entrepreneurial persistence arises from multiple predictors (Holland and Shepherd, 2013). As a critical personal trait among entrepreneurs, persistence has long been deemed contingent on characteristics such as personality (Baum and Locke, 2004; Caliendo et al., 2014; Markman et al., 2005; Patel and Thatcher, 2014; Van Scotter and Garg, 2019) as well as skills and knowledge (Freeland and Keister, 2016). Some researchers have assumed an environmental perspective in determining that entrepreneurial persistence is also bound to attributes such as the institutional environment (Hayward et al., 2006; Mujtaba et al., 2021), entrepreneurial adversity (Gong and Yang, 2022; Holland and Shepherd, 2013), professional networks (Mujtaba et al., 2021; Welter and Smallbone, 2011), and social support (Haines and Townsend, 2014; Zhang, 2018). More recent studies from a psychological point of view have concerned the mental aspects of entrepreneurial persistence. Factors such as one's need for achievement (Sabiu et al., 2018), expectations (Van Scotter and Garg, 2019), sense of belonging (Chen et al., 2021), psychological ownership (Silla, 2020), entrepreneurial passion (Chen et al., 2021; Elhakim, 2022), and anticipated regret (Huang et al., 2023) jointly shape entrepreneurial persistence.

The above studies mostly pertained to contexts outside tourism. The formation and variation of tourism entrepreneurs' persistence lacks thorough investigation. Relevant research has often revolved around tourism business launches to the neglect of sustainable enterprise development. As a typical community- and place-based business, tourism entrepreneurship transcends interpersonal relationships to encompass person—place bonds (especially an entrepreneur's ties to their hometown). Hometown identity, as fostered by Confucian culture, may mold people's entrepreneurial persistence. It is therefore necessary to examine the antecedents of entrepreneurial persistence in the Chinese setting. Findings are expected to enrich the theory of entrepreneurial persistence and unearth astute managerial suggestions.

### 2.2 Hometown identity

Hometown identity involves the emotional connections that people form with their hometowns throughout life (Hu et al., 2017). This concept has many names: “hometown sentiment” (Lv et al., 2022), “hometown ties” (Zhu et al., 2021), “birthplace bias” (Lindblom et al., 2018), and “hometown favoritism” (Do et al., 2017). It embodies a complex type of place identity, referring to individuals' or groups' interactions for purposes such as socialization (Ren et al., 2020), self-definition (i.e., via a specific place), and the development of societal roles (Proshansky et al., 1983). In contrast to generic place identity, hometown identity is usually constructed through personal growth. Individuals may come to prefer people and things associated with their hometowns, hence their willingness to devote time, money, and energy to protecting these cherished places (Halpenny, 2010). Hometown identity can greatly influence one's behavior: officials with a strong hometown identity tend to favor their hometowns when crafting policies (Huang et al., 2022); businessmen with a strong hometown identity normally invest in their hometowns to boost local economic development (Zhu et al., 2021); and residents with a strong hometown identity are inclined to exhibit environmentally friendly behavior (Ren et al., 2020).

People in Eastern Asian countries, which are dominated by Confucianism, are apt to possess a strong hometown identity; it contributes to a moral foundation. One's hometown identity in this case entails a cycle of “falling leaves and returning to roots” (落叶归根). Under this philosophy, people are branches growing from roots. One's prosperity comes from these roots, and one's mission is to strengthen their roots (Fei, 2019). Hometown identity also holds ethical implications, such as prioritizing familial stability and kinship. These actions reinforce families' ethical order and moral obligations while maintaining interpersonal harmony and advocating for peace and kindness toward one's neighbors (Wang and Yin, 2019). Despite hometown identity's role in informing behavior, few studies have investigated its impact on entrepreneurial persistence.

### 2.3 Theory of planned behavior (TPB)

The theory of planned behavior (TBP) is a classic theory in social psychology meant to explain decision making. It is also a highly representative research framework with respect to rational behavior. The TBP asserts that behavioral intentions guide one's actions; attitude, subjective norms, and perceived behavioral control affect these intentions (Ajzen, 1991). Attitude refers to one's positive or negative evaluations of certain behaviors (Ajzen, 1985). Subjective norms, also known as perceived social norms, are based on social pressure to engage in particular behavior (Ajzen, 1985). These norms conventionally manifest from so-called “significant others”: parents and other relatives, close friends, and classmates (Ajzen, 1991). Perceived behavioral control involves one's assessments of internal and external objective factors based on personal experience and the relative difficulty of executing a given behavior (Ajzen, 1991).

The TBP can forecast people's intentions and actions. This theory is therefore useful for predicting entrepreneurial intention and behavior (Kautonen et al., 2015). Researchers have scarcely applied this model to entrepreneurial persistence. The present study bridges this gap by expanding the TPB's applicability. Per this theory, the impact mechanism of entrepreneurial persistence is as follows: attitude, subjective norms, and perceived behavioral control jointly affect rural tourism business owners' entrepreneurial persistence. Attitude is the degree to which these professionals approve of such persistence. Subjective norms reflect outside pressure when business owners consider persisting in entrepreneurship; this pressure can come from groups such as relatives, friends, and acquaintances. Perceived behavioral control reflects rural tourism entrepreneurs' abilities to perceive and control factors that promote or hinder entrepreneurial persistence. This type of persistence concerns rural tourism entrepreneurs' intentions to persist in business ownership. The TPB is a well-established theory—attitude, subjective norms, and perceived behavioral control have been shown to significantly and positively affect people's behavioral intentions in different situations.

Notably, the TPB is a broad framework. Scholars may need to add concepts or relationships to improve its explanatory power (Ajzen, 1991). This theory is also not infallible. Although its three core elements (i.e., attitude, subjective norms, and perceived behavioral control) provide a basis for behavior, they do not necessarily *motivate* behavior (Bagozzi and Nataraajan, 2000). Thus, in light of this study's context, home identity is introduced into the TPB.

### 2.4 Hometown identity and entrepreneurial persistence: hypothesized model

#### 2.4.1 Hometown identity, attitude, and entrepreneurial persistence

Place identity theory suggests that hometown identity influences one's attitude and behavior toward the local community (Chen et al., 2023). Individuals may feel emotionally attached to their hometowns and thus consider their communities' economic factors and interests when making decisions (Lindblom et al., 2018). Hometown identity can also activate pro-social motivations and compel people to strive to benefit others out of respect for the hometown group's welfare (Ren et al., 2020; Lemée et al., 2019). As entrepreneurship can critically improve tourism destinations' competitiveness and boost employment opportunities (Mottiara et al., 2018), rural tourism entrepreneurs with a stronger hometown identity may be highly concerned about the community's wellbeing and possess a moral obligation to persist in entrepreneurship. We presume that rural tourism entrepreneurs with a firmer hometown identity will display pro-social motivations and be more attuned to hometown members' wellbeing. Therefore, in addition to seeking profits, rural tourism entrepreneurs with a stronger hometown identity are more likely to persist in entrepreneurship that will enhance others' wellbeing. The following hypotheses are put forth as a result:

H1: Hometown identity significantly and positively affects entrepreneurial persistence via attitude.
Specifically,
H1a: Hometown identity significantly and positively affects attitude.
H1b: Attitude significantly and positively affects entrepreneurial persistence.

#### 2.4.2 Hometown identity, perceived behavioral control, and entrepreneurial persistence

The theory of place identity further implies that this type of identity positively influences entrepreneurial self-efficacy; that is, the environment enables better self-efficacy (Yin and Zhou, 2023). A sense of belonging to one's local environment can increase one's confidence and abilities (Rob et al., 2015). Familiarity with and attachment to a place may also lead people to feel unique and to treat themselves well, thereby evoking feelings of distinctiveness, continuity, self-esteem, and self-efficacy (Wang et al., 2021). Hometown identity can amplify rural tourism entrepreneurs' self-efficacy. This outcome is analogous to perceived behavioral control (Ajzen, 1991). The following hypotheses are proposed accordingly:

H2: Hometown identity significantly and positively affects entrepreneurial persistence through perceived behavioral control.
Specifically,
H2a: Hometown identity significantly and positively affects perceived behavioral control.
H2b: Perceived behavioral control significantly and positively affects entrepreneurial persistence.

#### 2.4.3 Hometown identity, subjective norms, and entrepreneurial persistence

A hometown is more than merely a geographic location; it includes local humanities, the environment, and social psychological connotations (Yin and Chen, 2020). One's hometown identity can shape their social identity, namely their “self-conception as a group member” (Rob et al., 2015). Social identity theory maintains that people develop relationships within groups and use these memberships to distinguish themselves from people outside their groups (Dutot, 2020). Family, blood relatives, and clans have exemplified social groups in China's traditional rural society (Tian et al., 2022). Intuitively, the stronger one's hometown identity, the more attention they pay to their family, blood relatives, and clans. People with a stronger hometown identity should be more sensitive to these groups' opinions and usually seek to abide by subjective norms and persist in entrepreneurship. Stated formally:

H3: Subjective norms significantly and positively affect entrepreneurial persistence.
H4: The stronger a rural tourism entrepreneur's hometown identity, the more significantly subjective norms influence entrepreneurial persistence.

**Figure 1:**
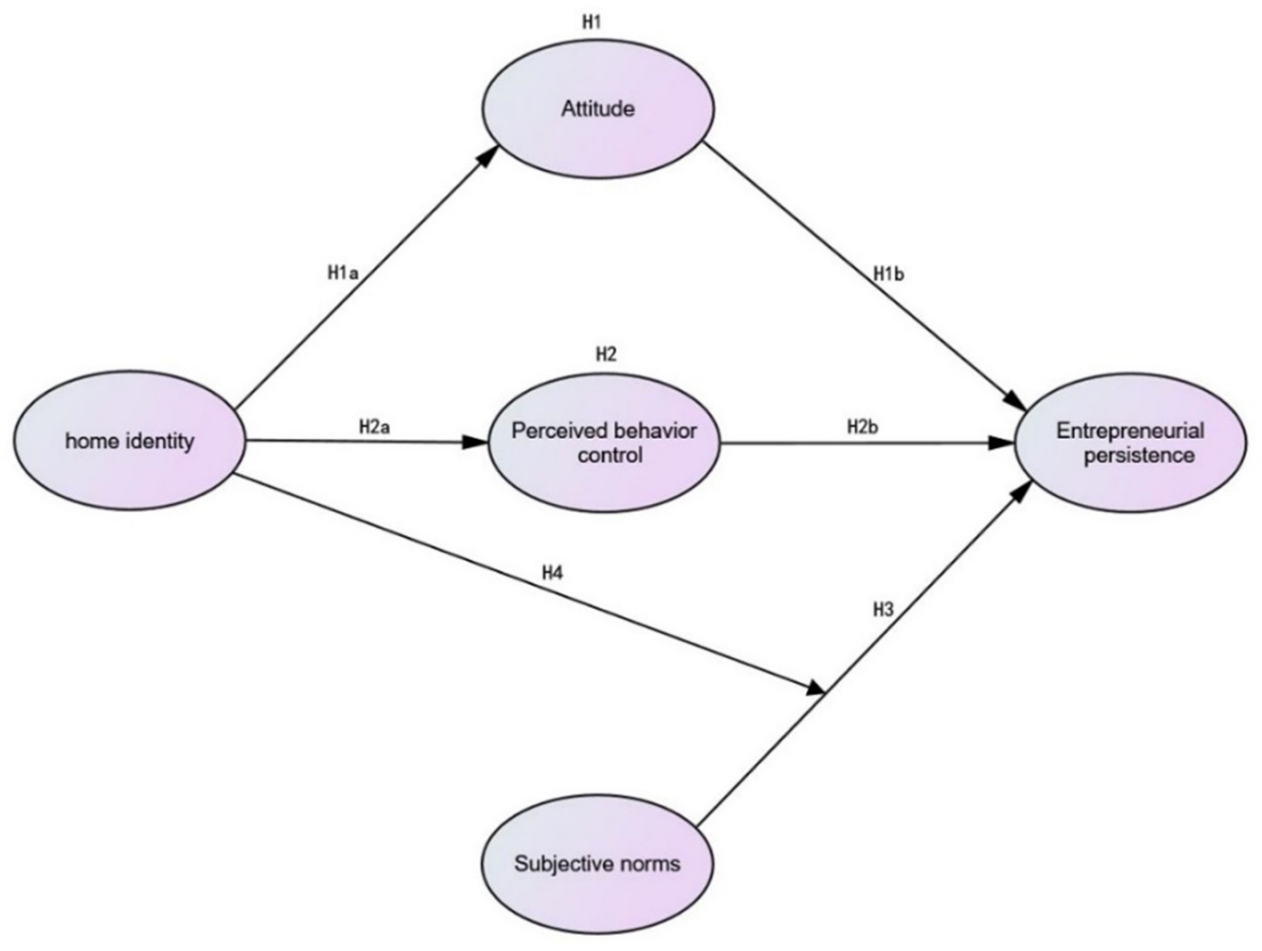
Conceptual framework.

The preceding hypothetical relationships are illustrated in Figure 1.

## 3 Methodology

The primary aim of this study is to delve into and validate the correlation between hometown identity and entrepreneurial persistence. Given the interpretive nature of this research, the utilization of quantitative research methods is deemed appropriate. Moreover, as rural tourism entrepreneurship is a global phenomenon, it is imperative to endorse the generalization of the research outcomes, extending them to a broader entrepreneurial cadre and various entrepreneurial environments. Notably, quantitative research supersedes qualitative research in terms of enabling the generalization of findings. Consequently, we employed a survey method based on questionnaires. These questionnaires offer quantitative evaluations and are subjected to analysis via numerical computations and statistical methodologies.

### 3.1 Instrument design and measurement

The survey used in this study comprised six sections: rural tourism entrepreneurs' characteristics (i.e., social and demographic attributes); hometown identity; attitude; subjective norms; perceived behavioral control; and entrepreneurial persistence. Relevant items were drawn from the literature. Hometown identity was measured using four items adapted from Xu et al. (2015). Attitude, subjective norms, and perceived behavioral control were assessed based on Ajzen's (1991) TPB measurement scale. Entrepreneurial persistence was evaluated via items from Samuel Adomako et al. (2016). All items were scored on a 5-point Likert-type scale (1 = *strongly disagree*, 5 = *strongly agree*). The items in the questionnaire are listed in Appendix A.

### 3.2 Study sites and data collection

**Figure 2:**
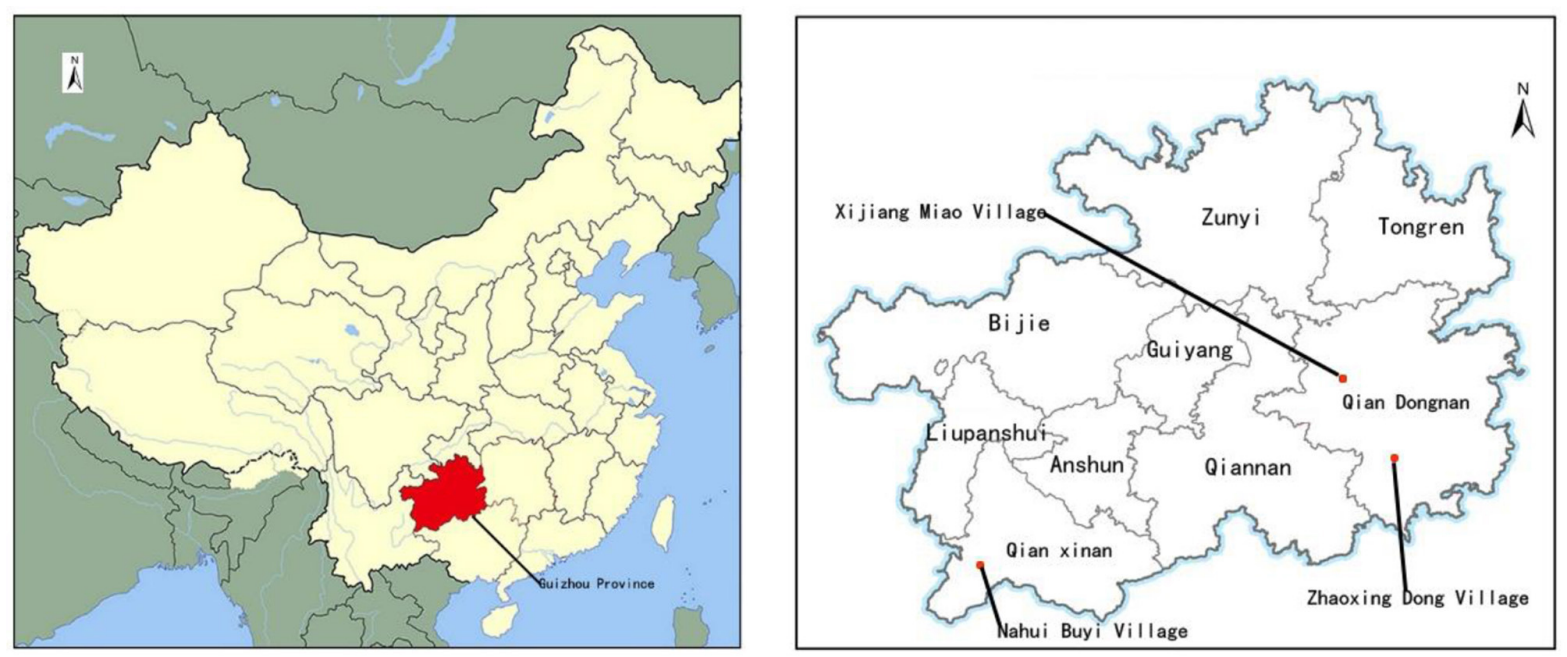
Location of Guizhou Province and three ethnic villages.

Xijiang Miao Village, Zhaoxing Dong Village, and Nahui Buyi Village in Guizhou Province, China, were selected to test our conceptual model. As a multi-ethnic country with 56 ethnic groups, China has Han Chinese comprising 91% of the total population, while ethnic minorities, including Miao, Dong, and Buyi, constitute 9% and are found in various regions. The strong tourism appeal of these culturally rich minority groups has led many ethnic villages to become prominent rural tourism destinations. These villages have preserved traditional culture and lifestyles, and local rural tourism entrepreneurs typically exhibit a strong hometown identity. Consequently, they tend to prioritize their cultural heritage and are more inclined to invest in the development of local rural tourism. Figure 2 shows the location of Guizhou Province and the three villages.

**Figure 3:**
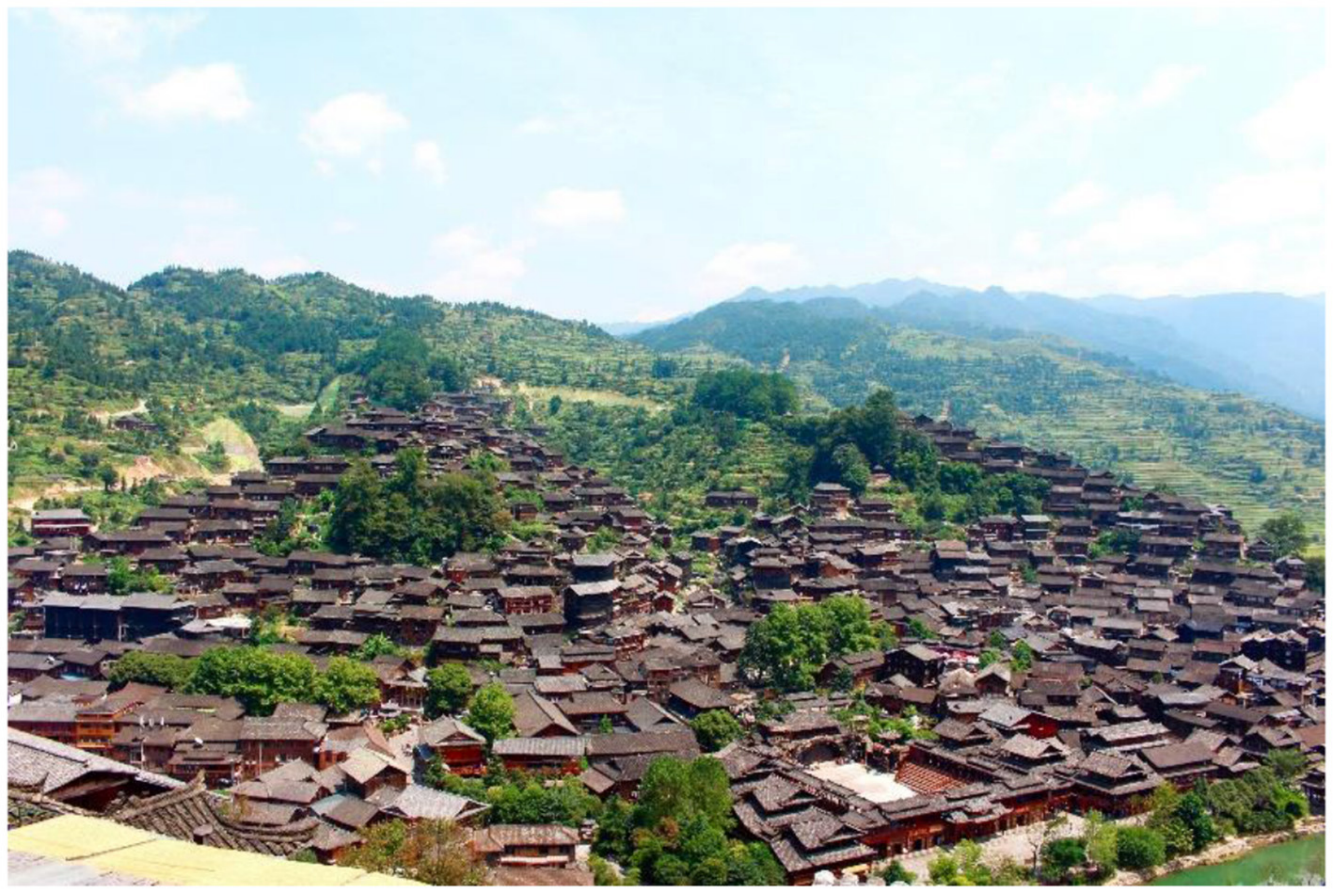
Xijiang Miao Village.

Xijiang Miao Village stands at the foot of Leigong Mountain in the northeastern part of Leishan County. This village is 36 km from the county seat and roughly 200 km from Guiyang, the capital of Guizhou Province. Xijiang Miao Village has 1,400 households and a population of 5,515. Among the 1,500 tourism entrepreneurs in the area, local entrepreneurs comprise approximately 61%. Since the Third Tourism Development Conference of Guizhou Province in 2008, the village's tourism industry has rapidly developed, establishing it as a globally recognized rural tourism destination. This growth has played a crucial role in advancing local poverty alleviation and economic enrichment. Figure 3 provides a comprehensive view of the village.

**Figure 4:**
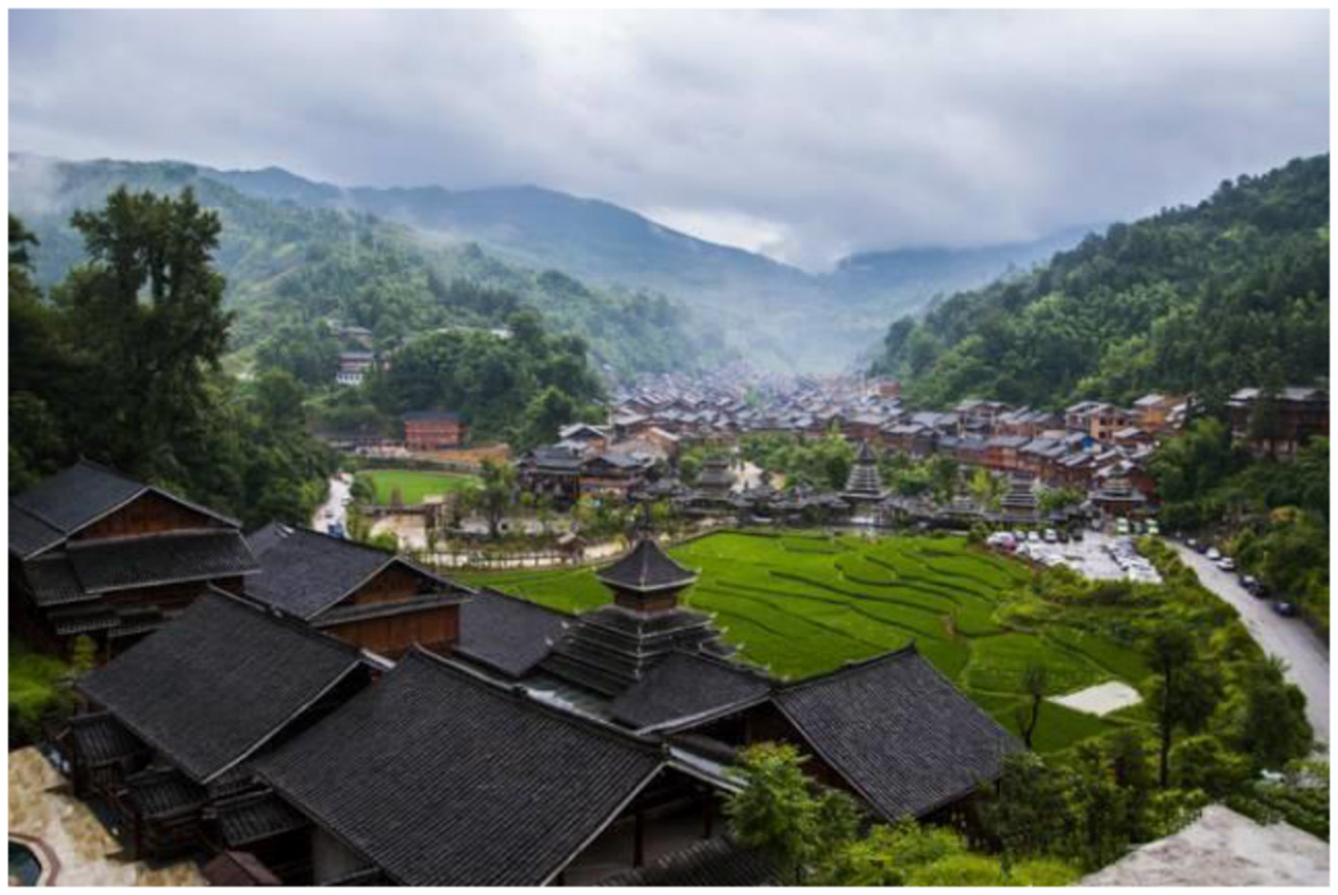
Zhaoxing Dong Village.

Zhaoxing Dong Village, in the southeastern part of Liping County, Guizhou Province, is one of China's largest Dong villages. This village is 70 km from the county seat and roughly 280 km from Guiyang, the capital of Guizhou Province. Zhaoxing Dong Village has 1,012 households and a population of 4,146. There are approximately 1,000 tourism entrepreneurs in Zhaoxing, with local entrepreneurs making up about 63% of this number. In 2007, the village was recognized by National Geographic magazine as one of the 33 most attractive tourist destinations in the world. Zhaoxing Dong Village has successfully carved out a unique development path, with culture as its foundation and tourism as its driving force, thereby infusing vitality into rural revitalization efforts. Figure 4 illustrates the village's landscape.

**Figure 5:**
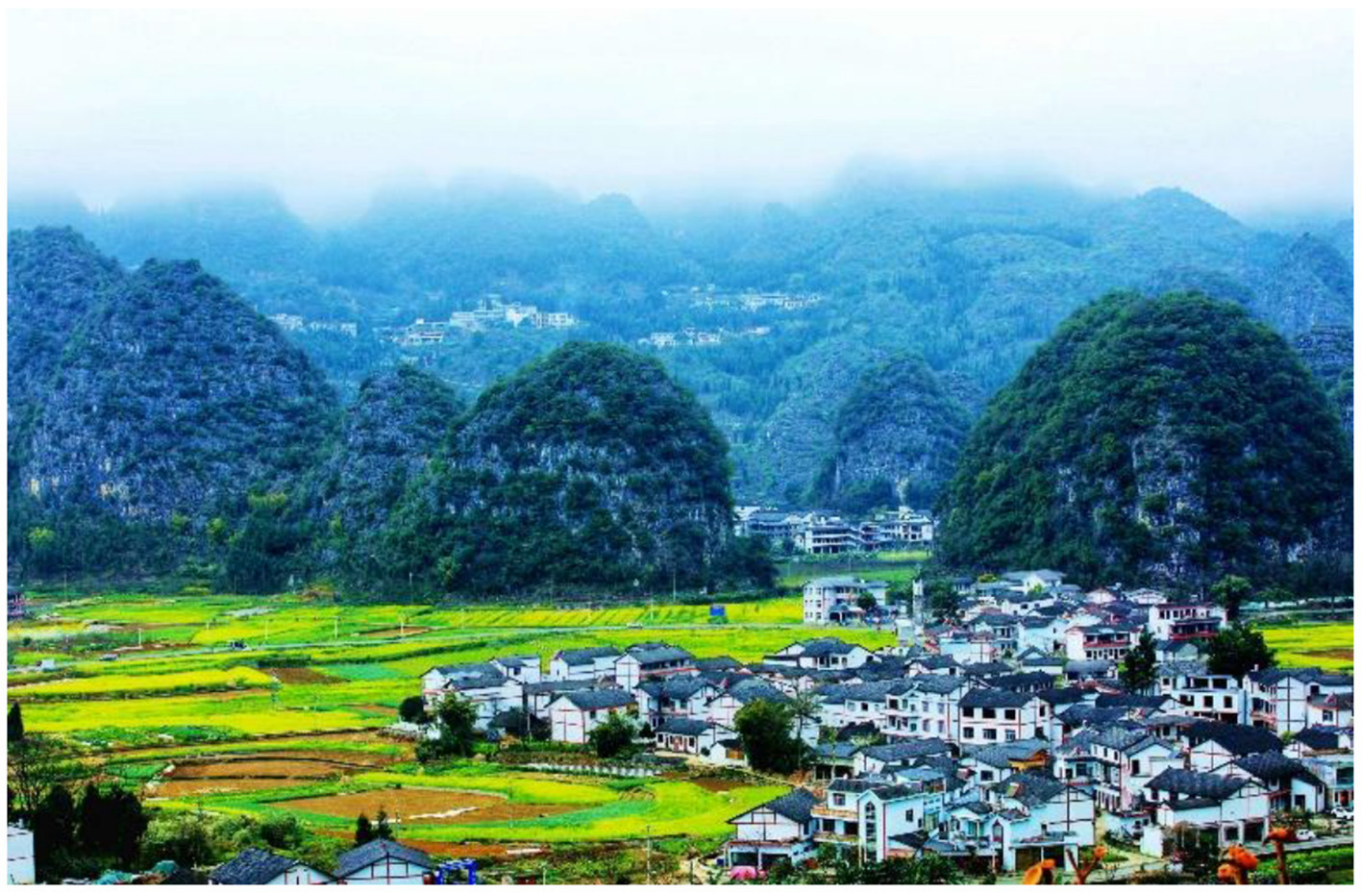
Nahui Buyi Village.

Nahui Buyi Village is a Buyi village and is in Xingyi City, Guizhou Province. It boasts rich ethnic customs and beautiful rural scenery at the Wanfenglin scenic spot, which sits 12 km from the urban district of Xingyi and about 300 km from the provincial capital. There are 450 households with a population of 1,950 in Nahui Buyi Village. The village has a pleasant climate, with an average annual temperature of 16–18°C, no severe cold in winter and no scorching heat in summer, making it a famous tourist resort. As of 2024, there are more than 300 rural tourism entrepreneurs here, of which local entrepreneurs account for about 56%. Tourism has become the pillar industry of the village, which has greatly contributed to the local socio-economic development. Figure 5 shows a snapshot.

The survey was conducted from November 25 to December 4, 2022, across three villages. Due to time and financial constraints, a convenience sampling strategy was employed, selecting tourism entrepreneurs non-randomly, as outlined by Robin and Haywantee (2012). According to Klein and Hall (1994), sample sizes should be at least 10 to 20 times the number of measurement items. The respondents consisted of locally born entrepreneurs. To ensure that the participants represented the target population, investigators verified the entrepreneurs' place of origin before commencing the survey. The target sample size was set at 500, which is more than 20 times the number of measurement items. To account for the expected recovery rate of 80–85% in similar surveys, 600 questionnaires were distributed: 300 in Xijiang Miao Village, 200 in Zhaoxing Dong Village, and 100 in Nahui Buyi Village, reflecting the number of tourism entrepreneurs in each area. A total of 552 surveys were returned, and after removing 58 invalid questionnaires, the valid sample comprised 494 surveys, resulting in an effective response rate of 82.3%.

### 3.3 Data analysis

Data were processed in AMOS 24.0 and SPSS 22.0. A descriptive analysis was initially performed to summarize the sample's demographic characteristics. Next, confirmatory factor analysis (CFA) was conducted to verify constructs' reliability and validity. Structural equation modeling (SEM) was then carried out to test direct effects. Finally, mediating effects were tested via the bootstrap method, and moderating effects were tested using the factor pairing method.

## 4 Results

### 4.1 Descriptive analysis

Table 1 presents an overview of respondents' demographics. Men accounted for 61.5% of the sample. Most respondents were young or middle-aged, between the ages of 30 and 60 (74.7%). Many possessed either a secondary school education (48.8%) or high school education (26.9%). More than half of respondents (59.3%) started their businesses more than 3 years ago. Collectively, over half of respondents' businesses involved tourism commodities (39.7%) and restaurants (35.4%). Two measures (i.e., skewness and kurtosis) were examined to determine data normality (Hair, 2014). As listed in Table 2, the absolute values of skewness did not exceed 3 and those of kurtosis did not exceed 10, satisfying the normality hypothesis (Kline, 2015).

**Table 1:** Demographic profile of respondents (*N* = 494).

**Gender**	***N***	**Percentage**	**Duration of entrepreneurship**	***n***	**Percentage**
Male	304	61.5	1 year	87	17.6
Female	190	38.5	1–2 years	52	10.5
**Age**			2–3 years	62	12.6
< 30	108	21.9	>3 years	293	59.3
30–40	141	28.5	**Business type**		
40–50	146	29.6	Hotels	78	15.8
50–60	82	16.6	Restaurants	175	35.4
>60	17	3.4	Commodities	196	39.7
**Education**			Entertainment	45	9.1
Secondary school	241	48.8			
High school	133	26.9			
College	118	23.9			
Post-graduate	2	0.4			

**Table 2:** Distribution of measurement items.

**Item**	**Mean**	**Std**.	**Skewness**	**Kurtosis**
HI1	4.08	0.911	−0.681	−0.234
HI2	4.07	0.942	−0.722	0.059
HI3	3.79	0.987	−0.191	−0.894
HI4	3.99	0.950	−0.507	−0.558
AT1	3.59	1.009	−0.644	0.108
AT2	3.43	1.024	−0.314	−0.286
AT3	3.23	1.083	−0.059	−0.573
SN1	3.46	0.906	−0.146	−0.227
SN2	3.35	0.927	−0.142	−0.028
SN3	2.91	1.031	0.338	−0.356
PBC1	3.29	1.013	−0.110	−0.500
PBC2	3.33	1.048	−0.001	−0.631
PBC3	3.17	1.015	0.235	−0.544
PBC4	3.60	1.024	−0.264	−0.385
EP1	2.51	1.054	0.433	−0.253
EP2	2.29	0.981	0.731	0.419
EP3	3.07	0.998	−0.184	−0.135

HI, hometown identity; AT, attitude; SN, subjective norms; PBC, perceived behavioral control; EP, entrepreneurial persistence.

### 4.2 Reliability and validity analysis

CFA was completed in AMOS 24.0 to establish the measurement model's reliability and validity. A model including all five variables (i.e., hometown identity, attitude, subjective norms, perceived behavioral control, and entrepreneurial persistence) demonstrated an acceptable fit: χ^2^/*df* = 3.569, *p* = 0.000; CFI = 0.942; RMR = 0.056; RMSEA = 0.072. Table 3 synthesizes these results. The average variance extracted (AVE) for each construct was > 0.5, indicating that all measures possessed sound convergent validity (Hair, 2014). Reliability was evaluated based on Cronbach's alpha (α) and composite reliability (CR) values, which were both higher than 0.7 (Nunnally and Bernstein, 1994). The questionnaire demonstrated discriminant validity because the square root of the AVE of each construct exceeded its highest association with the remaining constructs (Fornell and Larcker, 1981). Full results appear in Table 4.

**Table 3:** Reliability and convergent validity.

	**Loading**	**Cronbach's α**	**CR**	**AVE**
HI1	0.809	0.889	0.892	0.674
HI2	0.872			
HI3	0.874			
HI4	0.720			
AT1	0.943	0.890	0.897	0.744
AT2	0.787			
AT3	0.851			
SN1	0.575	0.771	0.788	0.560
SN2	0.845			
SN3	0.798			
PBC1	0.862	0.899	0.902	0.698
PBC2	0.894			
PBC3	0.848			
PBC4	0.728			
EP1	0.777	0.773	0.778	0.540
EP2	0.762			
EP3	0.659			

**Table 4:** Discriminant validity.

	**HI**	**AT**	**SN**	**PBC**	**EP**
HI	**0.821**				
AT	0.414	**0.863**			
SN	0.231	0.498	**0.748**		
PBC	0.125	0.328	0.422	**0.835**	
EP	0.225	0.493	0.473	0.341	**0.735**

Bold values on the diagonal indicate the square roots of AVE values.

### 4.3 Structural equation model

SEM was performed in AMOS 24.0 to test the research hypotheses. The bootstrap method was used to assess mediating effects (i.e., of attitude and perceived behavioral control on the relationship between hometown identity and entrepreneurial persistence). This approach does not need to consider whether the data are normally distributed, and its statistical effect is superior to the Sobel test and other methods (Hayes, 2014). Bias-corrected bootstrapping was carried out for repeated sampling (2,000 times) to evaluate mediating effects at a 95% confidence level.

**Figure 6:**
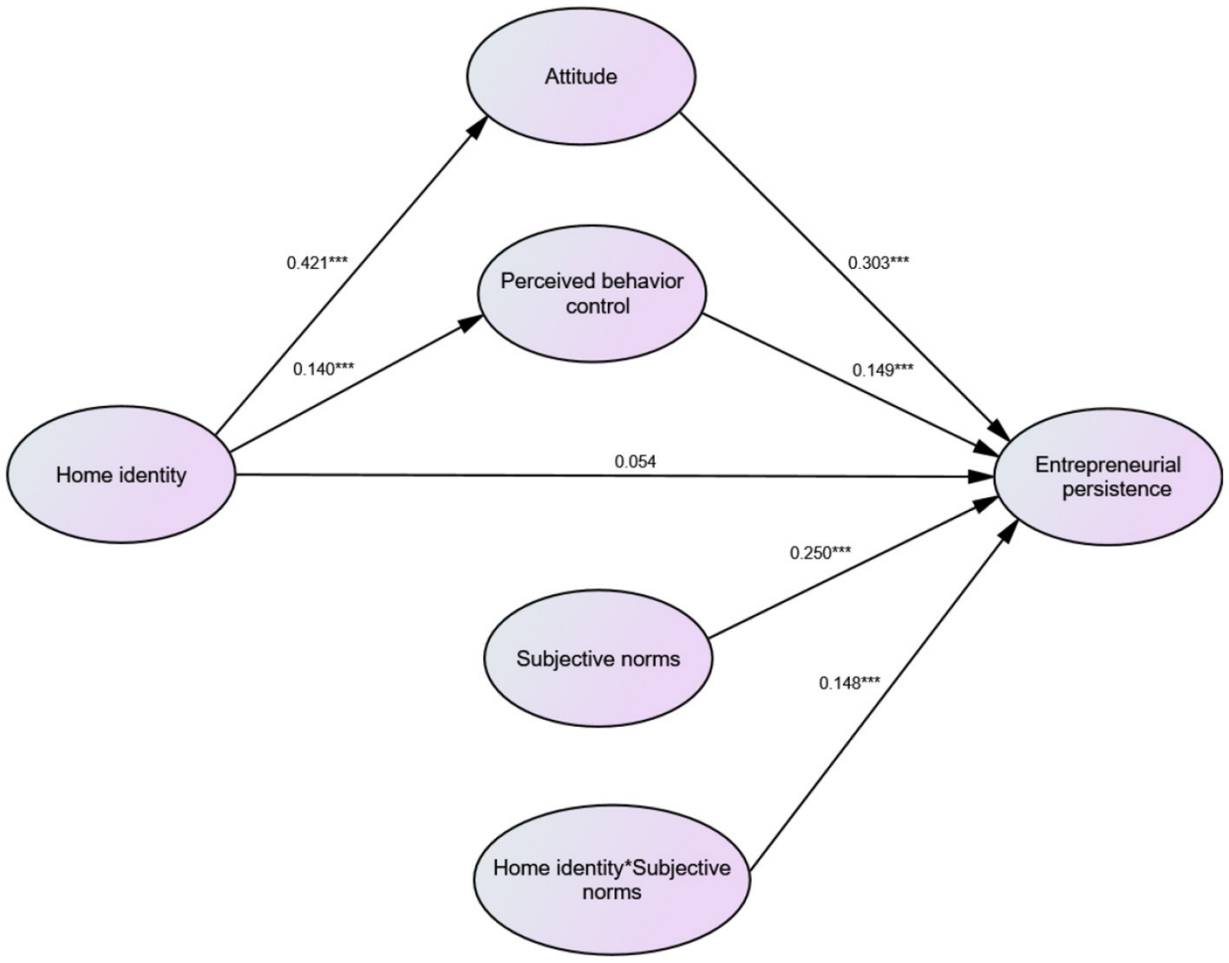
SEM results. ***p < 0.01.

Kenny and Judd's (1984) strategy was employed to verify the moderating impact of hometown identity. They recommended adding the interaction terms of the independent variables and moderator variables into the structural equation. A single-factor CFA was performed on subjective norms and hometown identity in accordance with Marsh's et al. (2004) paired product index technique. The three indices with the largest standardized factor loadings were chosen from the measurement items respectively associated with these two variables. Based on decentralizing these indices, and in line with the principle of matching factor loadings by size, the indices were matched (via similarly sized factor loadings) and multiplied together to obtain the measurement index of the interaction terms. The structural equation model was subsequently assembled in AMOS 24.0. The SEM results indicated an acceptable overall fit: χ^2^/*df* = 3.633, *p* = 0.000; CFI = 0.940; RMR = 0.079; RMSEA = 0.073. Figure 6 displays these results.

#### 4.3.1 Direct effects

As displayed in Table 5, HI had a significant effect on attitude (β = 0.421, *p* < 0.01), and attitude significantly influenced entrepreneurial persistence (β = 0.303, *p* < 0.01); H1a and H1b were thus supported. HI had a significant impact on perceived behavioral control (β = 0.140, *p* < 0.01), and perceived behavioral control played a significant role in entrepreneurial persistence (β = 0.149, *p* < 0.01); H2a and H2b were therefore supported. Subjective norms significantly influenced entrepreneurial persistence (β = 0.250, *p* < 0.01), lending support to H3.

**Table 5:** Direct effects test.

**Hypothesis**	**Predicted relationship**	**Standardized path loading**	**Standard error**	**Critical ratio**	***p***	**Result**
H1a	HI → AT	0.421	0.062	8.807	^***^	Supported
H1b	AT → EP	0.303	0.050	4.993	^***^	Supported
H2a	HI → PBC	0.140	0.056	2.899	^***^	Supported
H2b	PBC → EP	0.149	0.089	3.857	^***^	Supported
H3	SN → EP	0.250	0.048	2.865	^***^	Supported

^***^p < 0.01.

#### 4.3.2 Mediating effects

The test results in Table 6 indicate that the 95% confidence intervals of the mediating effects of attitude and subjective norms did not contain 0. Additionally, attitude and perceived behavioral control each mediated the relationship between hometown identity and entrepreneurial persistence. H1 and H2 were supported.

**Table 6:** Mediating effects and bias-corrected bootstrap test.

**Hypothesis**	**Predicted relationship**	**Effect**	***p***	**Bias-corrected (95%)**		**Result**
				**Lower**	**Upper**	
H1	HI → AT → EP	0.128	^***^	0.083	0.212	Supported
H2	HI → PBC → EP	0.021	^**^	0.003	0.055	Supported

^***^p < 0.01; ^**^p < 0.05.

#### 4.3.3 Moderating effects

Table 7 shows that the path coefficient of the interaction term of subjective norms and hometown identity on entrepreneurial persistence was 0.148 (*p* < 0.05). In other words, the moderating effect of hometown identity was significant, providing support for H4.

**Table 7:** Moderating effects test.

**Hypothesis**	**Predicted relationship**	**Standardized path loading**	**Standard error**	**Critical ratio**	***p***	**Result**
H4	Interaction → EP	0.148	0.056	2.596	^**^	Supported

^**^p < 0.05.

Interaction: the interaction term of subjective norms and hometown identity.

## 5 Discussion and conclusion

Entrepreneurial persistence is a function of various predictors (Holland and Shepherd, 2013). Despite efforts to pinpoint the determinants of persistence, relevant emotional facets and their effects on persistence are not yet fully understood. Most related studies have also involved a Western cultural background. The impact mechanisms of emotional aspects on Chinese entrepreneurs' persistence thus deserve a closer look. We investigated a crucial factor, rural tourism entrepreneurs' hometown identity, to determine its role in entrepreneurial persistence. Several findings warrant discussion.

First, in our case, rural tourism entrepreneurs' hometown identity positively affected their attitudes toward entrepreneurial persistence and indirectly influenced entrepreneurial persistence. As hometown identity is a complex form of place identity (Ren et al., 2020), this finding echoes the view that place identity influences one's attitudes and behaviors toward the local community (Chen et al., 2023; Hallak et al., 2012; Pretty et al., 2003). This result corresponds to the belief (Lewicka, 2005; Lindblom et al., 2018; Ren et al., 2020; Stedman, 2002; Huang and Du, 2022) that hometown identity may activate individuals' pro-social motivation and lead them to help others out of concern for the hometown group's welfare. Entrepreneurial persistence is key to promoting rural tourism destinations' sustainable development: this form of identity drives rural tourism business owners to persist in entrepreneurship to benefit residents.

Second, rural tourism entrepreneurs' hometown identity positively influenced perceived behavioral control and indirectly affected entrepreneurial persistence. Perceived behavioral control is similar to self-efficacy (Rob et al., 2015). Therefore, our outcome supports the argument (Yin and Zhou, 2023; Rob et al., 2015; Wang et al., 2021) that place identity has a significant and positive impact on entrepreneurial self-efficacy. Hometown identity is essentially conducive to enhancing rural tourism entrepreneurs' self-confidence.

Third, rural tourism entrepreneurs' hometown identity positively moderated the role of subjective norms on entrepreneurial persistence. Our finding is in accordance with the framing of hometown identity as one's in-group perceptions (Yin and Chen, 2020). This conceptualization carries ethical value implications: people aim to maintain a stable family unit and to reinforce kinship by strengthening the family's ethical order and moral obligations (Wang and Yin, 2019). Hometown identity also entails respect for the opinions of family, relatives, and friends in the rural Chinese context. We have extended relevant knowledge by proposing a moderating effect of hometown identity.

Fourth, these three findings further validate the Theory of Planned Behavior (TPB) and its relevance to entrepreneurship research and the study of persistence intentions. TPB posits that an individual's attitudes, subjective norms, and perceived behavioral control positively influence their behavioral intentions (Ajzen, 1991). In the context of this research, attitudes, subjective norms, and perceived behavioral control are shown to positively affect the entrepreneurial persistence of rural tourism business owners, thereby affirming the tenets of TPB. Additionally, our results corroborate the assertions of Kautonen et al. (2015) and Ahmed et al. (2024) that the theory effectively predicts entrepreneurial intentions and behaviors. Furthermore, the findings of this study illustrate the applicability of TPB in persistence intention research, aligning with the conclusions of Nathalie et al. (2018) and Kyung and Hee (2017).

Lastly, no significant direct relationship was observed between hometown identity and entrepreneurial persistence. Hometown identity instead demonstrated a significant indirect relationship with entrepreneurial persistence. Attitude and perceived behavioral control each mediated this relationship. In our context, rural business owners with a stronger hometown identity were more likely to persist in entrepreneurship. Positive emotions have been shown to permeate the entrepreneurial process and to encourage entrepreneurial persistence (Li et al., 2021). Earlier entrepreneurship studies identified emotional antecedents of entrepreneurial persistence such as entrepreneurial passion (Cardon and Kirk, 2015; Chen et al., 2021; Elhakim, 2022) and entrepreneurial wellbeing (Marshall et al., 2020; Wiklund et al., 2019). We have built on existing theory by illuminating hometown identity as a major emotional antecedent of entrepreneurial persistence in China's rural tourism industry.

Overall, we have confirmed the indirect impact of rural tourism entrepreneurs' hometown identity on entrepreneurial persistence. Yet this type of identity can shape entrepreneurial persistence through numerous means (e.g., attitude and perceived behavioral control). It can also moderate the impact of subjective norms on entrepreneurial persistence. Hometown identity additionally appears not to have a significant direct effect on entrepreneurial persistence. It is important to acknowledge that the selected research sites may influence the conclusions drawn from this study. This paper focuses on three ethnic minority villages in Guizhou, China, which remain relatively insulated and have preserved their traditional cultures and lifestyles. Local entrepreneurs place significant value on these traditions and exhibit strong emotional ties to their villages. These connections contribute to their willingness to engage in entrepreneurship aimed at fostering local development. The findings of this study underscore the role of hometown identity in promoting entrepreneurial persistence. Future research should expand the sample sites to enhance the validity of these results.

## 6 Implications and limitations

### 6.1 Theoretical implications

Entrepreneurial persistence among rural tourism business owners is vital to destinations' sustainable development in China. Clarifying the mechanisms behind entrepreneurial persistence is beneficial for both entrepreneurs and rural destinations. Our proposed framework of entrepreneurial persistence, based on hometown identity and the TPB, complements the extant literature: we introduced hometown identity into this research stream. Entrepreneurial persistence resulted from the impacts of hometown identity on attitude, subjective norms, and perceived behavioral control, revealing a novel interpretation of this construct. Emotional factors have been largely ignored in related work; however, entrepreneurial persistence is an emotional process (Lv et al., 2017; Shepherd, 2003). Our framework deepens relevant understanding, especially within Chinese culture.

Second, we have provided fresh insight into how rural tourism business owners persist in entrepreneurship. Scholars have primarily considered tourism enterprises' birth and survival. Less is known about the psychological process underpinning entrepreneurial persistence. We have also shown how the TPB model applies to entrepreneurial persistence. This theory has been verified in multiple settings but has certain weaknesses. Although attitude, subjective norms, and perceived behavioral control generally underlie intention and behavior, they do not drive behavior by themselves (Bagozzi and Nataraajan, 2000). We incorporated hometown identity into the TPB and uncovered motivations for attitude and perceived behavioral control. In addition, although this model is fairly common in research, few studies have applied it to tourism entrepreneurship. We have thus extended its use. Similarly, we integrated place identity theory in tourism entrepreneurship research. Hometown identity is related to place identity (Ren et al., 2020), a concept which has appeared frequently in tourist behavior studies to describe people–place bonds. It is comparatively rare in the tourism entrepreneurship domain (Hallak et al., 2012, 2013; Rob et al., 2015). Our work sheds additional light on the theory.

### 6.2 Managerial implications

The findings of this study highlight the importance of entrepreneurial persistence for the development of rural tourism, providing destination managers with valuable insights. By revisiting the theme of entrepreneurial persistence, this research offers a fresh perspective on rural tourism destination management and related policy development. It elucidates the mechanisms driving entrepreneurial persistence among rural tourism entrepreneurs from an affective standpoint, aiming to empower destination managers to foster this persistence among entrepreneurs. Authorities should emphasize the cultivation of a strong hometown identity among entrepreneurs when formulating and implementing destination-related policies to ensure the sustainable development of rural tourism.

First, rural destination managers must prioritize the protection of the cultural environment and the preservation of local cultural memory to stimulate the cultural identity of tourism entrepreneurs, thereby enhancing their hometown identity. This involves developing policies that safeguard traditional buildings, festivals, and customs to ensure the transmission of these cultural elements. Additionally, managers should organize cultural activities that deepen entrepreneurs' understanding of the countryside's history, customs, and lifestyle, while also inviting local residents to participate to promote interaction and strengthen the entrepreneurs' sense of belonging.

Second, rural destination managers can optimize the rural entrepreneurial environment by providing support services that enhance entrepreneurs' sense of social support and, consequently, their hometown identity. For instance, they can organize skills training and workshops to improve entrepreneurs' professional capabilities, as well as regular symposiums and sharing sessions where entrepreneurs can discuss their experiences, challenges, and solutions.

Finally, managers should promote collaboration among entrepreneurs to foster a sense of teamwork and enhance their hometown identity. This can be achieved by encouraging entrepreneurs to initiate or participate in cooperative projects, such as the joint development of unique tourism products, which can promote collaboration and the realization of shared interests. Additionally, managers can facilitate joint marketing efforts, including collaborative discounts, packages, or co-organized promotional events.

### 6.3 Limitations

Several limitations of this study leave room for future work. First, we referred to cross-sectional data, whereas entrepreneurial persistence is likely dynamic. Longitudinal investigations will better capture this process. Second, respondents were limited to entrepreneurs from rural tourism destinations in Guizhou, China. Scholars should recruit diversified samples to further validate our framework. Third, entrepreneurial persistence manifests from the interplay between people and the environment. We specifically addressed the roles of hometown identity, attitude, subjective norms, and perceived behavioral control on entrepreneurial persistence. Subsequent work should account for other environmental and personal factors to construct a more comprehensive model.

## Data Availability

The raw data supporting the conclusions of this article will be made available by the authors, without undue reservation.

## Appendix

**APPENDIX A:** Questionnaire items in measurement scales.

**Construct**	**Code**	**Measurement item**	**Source**
Hometown identity	HI1	I feel my hometown is a part of me.	Xu et al., 2015
	HI2	My hometown is very special to me.	Xu et al., 2015
	HI3	I identify strongly with my hometown.	Xu et al., 2015
	HI4	I am very attached to my hometown.	Xu et al., 2015
Attitude	AT1	Persistence in entrepreneurship is very pleasant behavior.	Ajzen, 1991
	AT2	Persistence in entrepreneurship is very wise behavior.	Ajzen, 1991
	AT3	Persistence in entrepreneurship is a very beneficial behavior.	Ajzen, 1991
Subjective norms	SN1	Relatives approve of my persistence in entrepreneurship here.	Ajzen, 1991
	SN2	Friends agree that I should persist in entrepreneurship here.	Ajzen, 1991
	SN3	Acquaintances other than friends and relatives approve of my persistence in entrepreneurship here.	Ajzen, 1991
Perceived behavioral control	PBC1	I can persist in entrepreneurship here as long as I want to	Ajzen, 1991
	PBC2	I have the money, time and ability to stay here and persist in entrepreneurship.	Ajzen, 1991
	PBC3	I think it is entirely up to me to decide whether or not to persist in entrepreneurship.	Ajzen, 1991
	PBC4	I don't think it's difficult to stay in business here.	Ajzen, 1991
Entrepreneurial Persistence	EP1	When others give up entrepreneurship, I will still choose to stick with it.	Adomako et al., 2016
	EP2	Even if others oppose me, I will continue to work hard.	Adomako et al., 2016
	EP3	No matter how challenging my entrepreneurship may be, I will never give up.	Adomako et al., 2016
